# Rewetting does not return drained fen peatlands to their old selves

**DOI:** 10.1038/s41467-021-25619-y

**Published:** 2021-10-05

**Authors:** J. Kreyling, F. Tanneberger, F. Jansen, S. van der Linden, C. Aggenbach, V. Blüml, J. Couwenberg, W-J Emsens, H. Joosten, A. Klimkowska, W. Kotowski, L. Kozub, B. Lennartz, Y. Liczner, H. Liu, D. Michaelis, C. Oehmke, K. Parakenings, E. Pleyl, A. Poyda, S. Raabe, M. Röhl, K. Rücker, A. Schneider, J. Schrautzer, C. Schröder, F. Schug, E. Seeber, F. Thiel, S. Thiele, B. Tiemeyer, T. Timmermann, T. Urich, R. van Diggelen, K. Vegelin, E. Verbruggen, M. Wilmking, N. Wrage-Mönnig, L. Wołejko, D. Zak, G. Jurasinski

**Affiliations:** 1 University of Greifswald, partner in the Greifswald Mire Centre, Greifswald, Germany; 2 University of Rostock, Rostock, Germany; 3 KWR Water Research Institute, Nieuwegein, The Netherlands; 4 BMS-Umweltplanung in association with University of Bremen, Osnabrück, Germany; 5 University of Antwerp, Antwerp, Belgium; 6 University of Warsaw, Faculty of Biology, Warsaw, Poland; 7 Landkreis Vorpommern-Greifswald, Anklam, Germany; 8 Zentrum für Umwelt und Kultur Benediktbeuern e.V., Benediktbeuern, Germany; 9 Christian-Albrechts-Universität Kiel, Kiel, Germany; 10 Hochschule für Wirtschaft und Umwelt Nürtingen-Geislingen, Nürtingen-Geislingen, Germany; 11 Regional administration Havelland, Nauen, Germany; 12 Stiftung Umwelt und Naturschutz M-V, Bugewitz, Germany; 13 Humboldt-Universität zu Berlin, Berlin, Germany; 14 Institut für Landschaftsökologie und Naturschutz GmbH, Greifswald, Germany; 15 Thünen-Institute of Climate-Smart Agriculture, Braunschweig, Germany; 16 BNL Vegelin; Groß Polzin, Greifswald, Germany; 17 West Pomeranian University of Technology Szczecin, Szczecin, Poland; 18 Leibniz-Institute of Freshwater Ecology and Inland Fisheries, Berlin, Germany; 19 Aarhus University, Aarhus, Denmark

**Keywords:** Ecosystem ecology, Plant ecology, Biogeochemistry

## Abstract

Peatlands have been drained for land use for a long time and on a large scale, turning them from carbon and nutrient sinks into respective sources, diminishing water regulation capacity, causing surface height loss and destroying biodiversity. Over the last decades, drained peatlands have been rewetted for biodiversity restoration and, as it strongly decreases greenhouse gas emissions, also for climate protection. We quantify restoration success by comparing 320 rewetted fen peatland sites to 243 near-natural peatland sites of similar origin across temperate Europe, all set into perspective by 10k additional European fen vegetation plots. Results imply that rewetting of drained fen peatlands induces the establishment of tall, graminoid wetland plants (helophytisation) and long-lasting differences to pre-drainage biodiversity (vegetation), ecosystem functioning (geochemistry, hydrology), and land cover characteristics (spectral temporal metrics). The Paris Agreement entails the rewetting of 500,000 km^2^ of drained peatlands worldwide until 2050-2070. A better understanding of the resulting locally novel ecosystems is required to improve planning and implementation of peatland rewetting and subsequent management.

## Abstract (web summary)

Whether rewetting leads to effective restoration of drained peatlands is unclear. Here the authors analyse a large number of near-natural and rewetted fen peatland sites in Europe, finding persistent differences in plant community composition and ecosystem functioning, and higher variance in the restored sites.

## Introduction

Already in 1658, the world’s first scientific book on peatlands^1^ included a chapter on peatland restoration and addressed the question of their long-term use. However, centuries of peatland destruction followed and large-scale restoration of drained peatlands is just about to begin^2^. Despite some progress in understanding ecological functioning of rewetted peatlands^3–5^, the early warning from 1764 that restoration success may be slow^6^ can only now be quantified at a substantial amount of rewetted sites, across continental scales and multiple ecosystem functions.

Intact peatlands provide numerous ecosystem services. They store huge amounts of carbon (600 ± 100 Gt^7^; thus 30% of the global soil carbon is found on only 3% of the global land^5,8^), they regulate water quality and quantity, and they harbor highly specialized biota^9–12^. Approx. 500,000 km², i.e., 10–15% of the current peatland area, have been drained for agriculture, peat extraction and forestry, historically mainly in temperate and boreal regions, but more recently also in the tropics^13^. Drainage of peatlands impairs their ecosystem service provisioning. Peat oxidation in drained peatlands is responsible for about 5% of the global anthropogenic greenhouse gas (GHG) emissions^12,14^. Peat mineralization leads to ongoing land subsidence^15^ and turns drained peatlands into sources of nutrients, causing eutrophication of downstream surface and groundwater^16^. A fundamental distinction is commonly made between bogs that are solely fed by rainwater, and fens that also receive ground- and/or surface water that has been in contact with the mineral soil or bedrock^10^. Fens cover more than half of Europe’s peatland area^10^. Especially temperate fens, which often become nutrient-rich and productive upon drainage, have been drained for agriculture^10^. Consequently, many temperate fen peatland species are globally endangered because of habitat loss^5,17,18^.

Rewetting drained peatlands can strongly reduce or stop net carbon loss immediately^14^ and may lead to new carbon sequestration^19,20^. In contrast, continued emissions from drained peatlands may comprise 12–41% of the GHG emission budget until 2100 for keeping global warming below +1.5 to +2 °C^21^. Compliance with the Paris Agreement implies carbon neutrality by 2050–2070^22^ meaning that until then the 500,000 km² of drained peatlands need to be rewetted, i.e., on average over one million hectares per year. The 2021–2030 UN Decade of Ecosystem Restoration has to meet this challenge.

However, rewetting might not restore natural conditions promptly or even within decades, in particular in severely disturbed and long-drained temperate fens (i.e., groundwater-fed peatlands), where oxidation during drainage has altered peat physical parameters and has led to, for instance, increased bulk density and decreased porosity, hydraulic conductivity and storativity^23^, leading to stronger water table fluctuations. Periodic or episodic inundation can provoke peaks of methane emissions^24,25^. Furthermore, nutrient availability after rewetting is considerably higher than in natural peatlands, mainly because of peat mineralization and fertilization while the peatland was drained^4,26^, and because of mobilization of phosphorus upon rewetting^27^. Consequently, microbial^4^ and plant^3^ communities show prompt recovery towards their pre-drainage status only in previously weakly disturbed ecosystems.

**Fig. 1:**
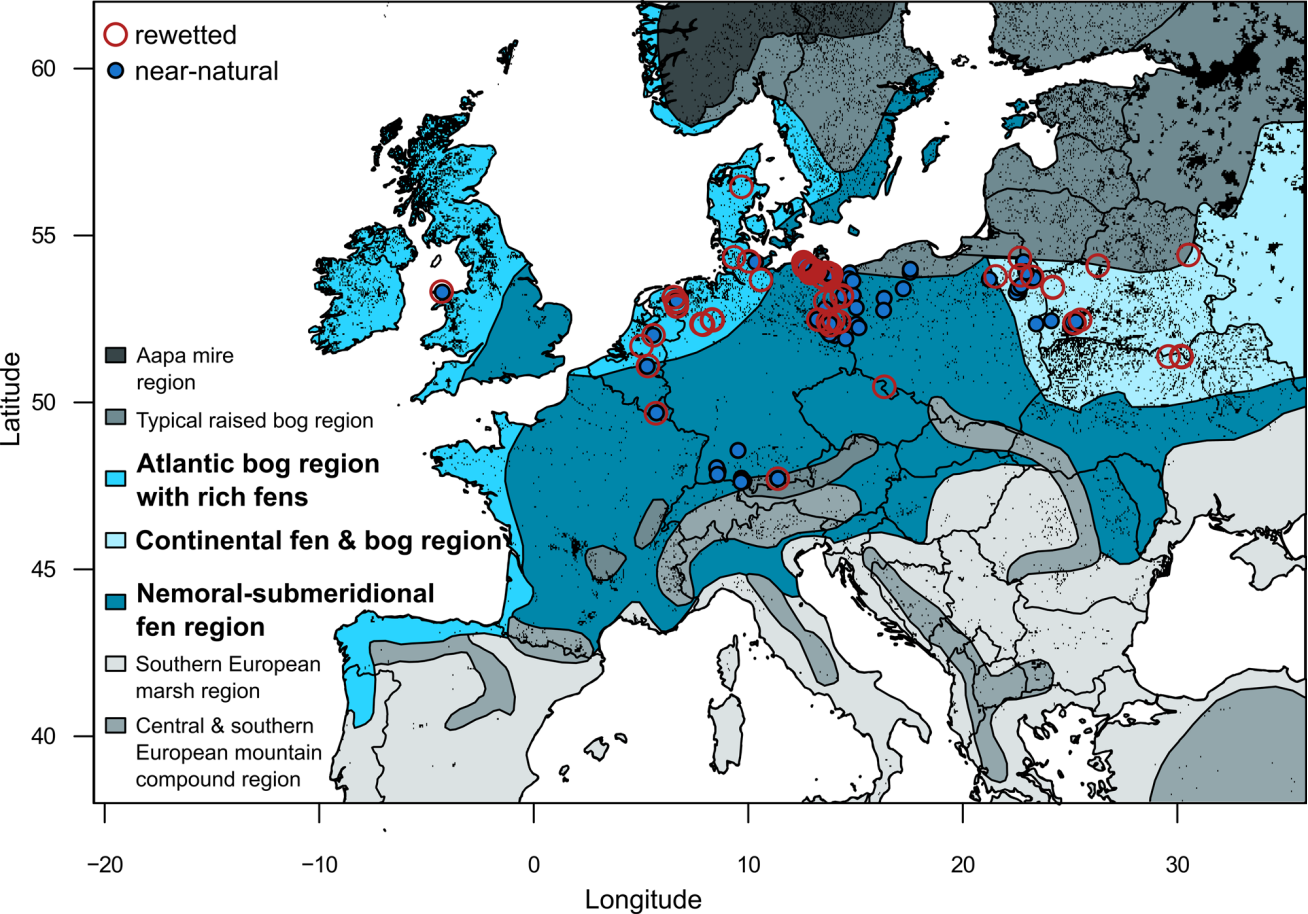
Map of the study sites contrasting rewetted sites (open red symbols) and near-natural sites (filled blue). We studied temperate fen peatlands, which occur mainly in the mire regions according to Joosten et al.^10^ depicted in shades of blue and set off in bold in the legend. Peatland occurrence according to the Peatland Map of Europe^53^ is shown in black.

Here, we compare 320 rewetted fen sites with 243 near-natural sites of similar origin from the major fen peatland regions of Europe (Fig. 1), which are further set into perspective by additional 10k vegetation plots, randomly chosen from >90k plots of European fen vegetation^18,28^ to quantify restoration success in terms of biodiversity (vegetation), ecosystem functioning (hydrology, geochemistry), and land cover characteristics. Our data imply that rewetting drained fens induces the establishment of tall, graminoid wetland plants, i.e., a helophytisation, with no trend back to their former biodiversity and ecosystem functioning for at least several decades in more than half of the sites. A better understanding of biodiversity and ecological functions of these locally novel ecosystems is urgently required to assess restoration success and to improve planning and implementation of peatland rewetting and the subsequent management.

## Results and discussion

### Rewetted peatlands differ in their biodiversity and ecosystem functioning from near-natural peatlands

**Fig. 2:**
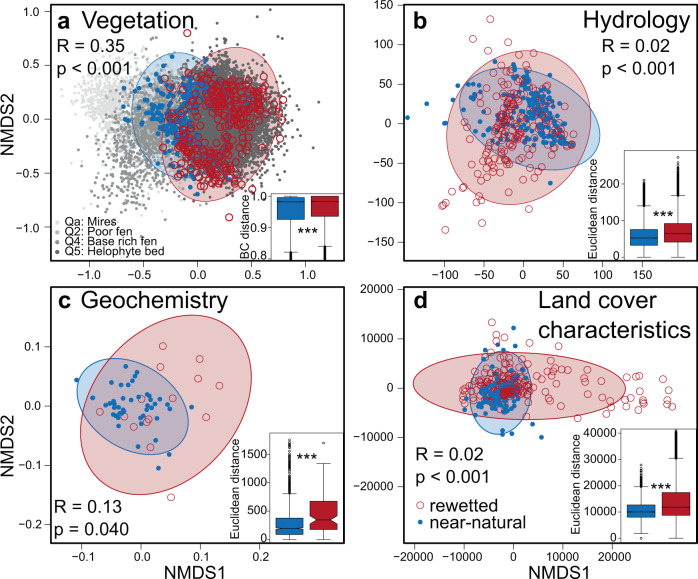
Rewetted temperate fen peatland sites (open red) are more variable than near-natural sites (filled blue) and many rewetted sites are outside the near-natural range of variation. Ordination (Non-metrical multidimensional scaling; NMDS) and analysis of similarity (ANOSIM) with its respective *R* value and *p* value provided in the figures. Dissimilarity is measured as Bray–Curtis distance for (**a**) vegetation based on 539 plant species (*n*_rewetted_ = 320, *n*_near-natural_ = 243), and Euclidean distance for (**b**) hydrology (based on annual median water table, annual minimum water table, annual maximum water table, annual amplitude of water table; *n*_rewetted_ = 320, *n*_near-natural_ = 243), (**c**) geochemistry (based on pH of pore water, electrical conductivity of the pore water in the top soil layer, bulk density, organic matter content of the top layer; *n*_rewetted_ = 16, *n*_near-natural_ = 47) and (**d**) land cover characteristics measured as 208 spectral-temporal metrics of high resolution optical Earth Observation data (*n*_rewetted_ = 258, *n*_near-natural_ = 114). The ellipses display 95%CI for each group. Sample size differs for the four response clusters according to data availability. The vegetation data are set into perspective against 10k vegetation plots randomly selected from > 90k vegetation plots from Europe of the displayed EUNIS-classes^28^ covering fen vegetation indicating the broad coverage of our near-natural sites. Note that no information about rewetting status is available for these background data. For vegetation, 63% of the rewetted sites are located outside the 95% confidence ellipse of the near-natural sites, for geochemistry 44%, for hydrology 20% and for spectral-temporal metrics 21%, respectively. Final stress of the two-dimensional ordinations are 0.16 for vegetation, 0.02 for hydrology, 0.04 for geochemistry, and 0.12 for land cover characteristics. The inserted boxplots display the median, quartiles (box), 1.5-fold quartile distance (whiskers) and extremes beyond the whiskers of all pairwise distances for rewetted sites in red and near-natural sites in blue (**a** and **b**: *n*_rewetted _= 51040, *n*_near-natural _= 29403; **c**: *n*_rewetted _= 120, *n*_near-natural _= 1081; **d**: *n*_rewetted _= 33,135, *n*_near-natural _= 6441). Significance of difference in mean values between rewetted and near-natural was evaluated by a permutation procedure as described in the methods section (***p < 0.001 for all four panels).

Rewetted fen peatlands differ in biodiversity and ecosystem functioning from near-natural peatlands, in particular regarding plant community composition and geochemistry (Fig. 2a, c). Smaller, but still highly significant differences were detected in hydrology and in land cover characteristics (Fig. 2b, d). The vegetation of the rewetted sites was furthermore less diverse at the plot scale across the range of Hill’s diversity numbers (Supplementary Fig. 1; Shannon diversity: 1.46 ± 0.04 (mean ± SE) in rewetted sites; 1.75 ± 0.04 in near-natural sites; *p*_*t.test*_ < 0.001).

**Fig. 3:**
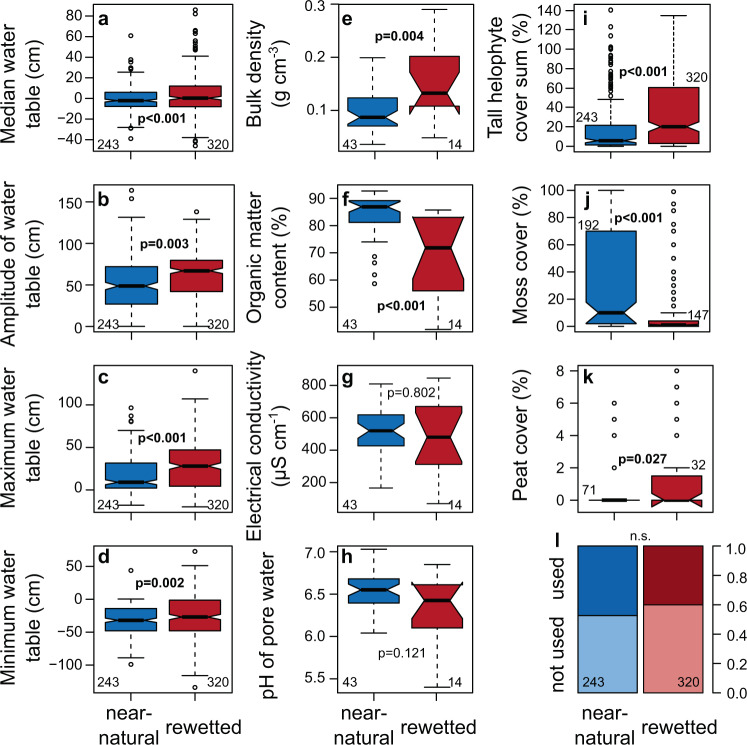
Rewetted fen peatlands differ in relevant ecological parameters from near-natural fen peatlands. Comparison between rewetted (red) and near-natural (blue) sites for all parameters used in the analyses for hydrology (**a**–**d**) and for geochemistry (**e**–**h**) alongside selected other relevant parameters (**i**–**m**). ‘Tall helophyte cover sum’ (**i**) is the sum over all tall helophyte species cover values according to Chytrý et al.^28^. Asterisks indicate significance of differences according to two-sided Wilcoxon-rank test for numerical and Chi² test for factorial response parameters (***p < 0.001; **p < 0.01; *p < 0.05). n per group is provided as numbers next to the boxplots, *n* differs between parameters as data was not available for all sites and all parameters, respectively. The boxplots display the median, quartiles (box), 1.5-fold quartile distance (whiskers) and extremes beyond the whiskers.

The differences in plant community composition between rewetted and near-natural sites are frequently linked to a helophytisation, i.e., the shift to a dominance of tall, graminoid wetland plants, as indicated by significant preferences of tall helophytes such as *Typha latifolia* or *Phalaris arundinacea* for the rewetted sites (see Indicator Species Analysis in Supplementary Data 2), by a higher prevalence of the EUNIS 2020 habitat type^28^ “tall helophyte bed” (relative frequency in rewetted peatlands: 25.5% versus 6.2% in near-natural peatlands; Chi²: *p* < 0.001; Supplementary Table 1), and by a 66% higher cover sum of tall helophyte species in rewetted as compared to near-natural sites (Fig. 3i). This helophytisation has implications beyond biodiversity as plant species composition affects carbon cycling by litter quality (e.g., polyphenol content^29^), root exudates, and production and consumption of carbon in the rhizosphere, both of which impact gaseous emissions. The tall helophytes are highly conductive for gases due to their aerenchyma, which may increase^30^ or suppress^31^ methane emissions. Brown mosses with high peat formation potential^32^ are largely absent from rewetted peatlands dominated by helophytes (Fig. 3j and Supplementary Data 2). While rewetting clearly reduces carbon emissions by inhibiting peat mineralization^14,33^, comparative analyses between the resulting vastly contrasting vegetation types are required to assess the total greenhouse gas effects of rewetting.

The helophytisation after rewetting is presumably caused by altered water table dynamics and geochemistry of rewetted peatlands. Peat mineralization, consolidation and compaction during the drained phase cause subsidence, leading to inundation after rewetting (mean median water table being 5.0 cm above surface in rewetted sites versus 1.5 cm below surface in near-natural sites and maximum water table differing by 74%; Fig. 3a, c). Peat mineralization is reflected in the lower organic matter content of the top soil layer (−18%) and in the higher bulk density (+61%; Fig. 3f, e), both of which decrease porosity and hydraulic conductivity^23^, provoking larger water table amplitudes (+15%) in rewetted sites (Fig. 3b). To sum up, inundation and eutrophic conditions promote tall helophytes, and these suppress low vascular plants and mosses by light competition^34^.

Land cover characteristics, i.e., the 208 metrics of the spectral-temporal variability of land surfaces in Earth observation time series^35^, also show differences between rewetted and near-natural peatlands (Fig. 2d), mainly attributed to high variety in the modified normalized difference water index (*MNDWI*) and the near infrared reflectance (*nIR*) for rewetted surfaces (Supplementary Table 2). High *MNDWI* values relate to a high share of open water in the pixels, *nIR* values correspond to vegetation density, vigor or type and are lowest for open water. Thus, both metrics relate to differences of the water regime for the vegetated surfaces. The dissimilarity between land cover characteristics in rewetted sites and near-natural sites hints at different pathways after rewetting. Environmental conditions such as hydrology, (chance) priority effects of colonizing species^36^ or former management - the rewetted sites did not differ from near-natural sites in share of unused sites after rewetting (Fig. 3l) - may determine the outcome.

A basic feature of natural peatlands is their stability, which is commonly attributed to a high degree of self-regulation, for instance with regards to buffering hydrological extremes by surface oscillation^37^. Further, stable, azonal species compositions indicate some degree of independence from zonal climate conditions^38^. Plant community composition and carbon dynamics in rewetted peatlands, however, are highly responsive to weather extremes^39^, hinting at a reduced stability. In our dataset, rewetted peatlands in all four response clusters were generally more variant than near-natural peatlands (Fig. 2 insets). This variance of rewetted peatlands may have resulted from a poorly restored hydrology on the landscape level and potentially impairs their ecosystem functioning and service provisioning. For instance, reduced oscillation capacity creates both times with water tables dropping low and inducing carbon dioxide emissions due to increased mineralization^40^, and times of inundation with increased methane emissions^24,25^.

### No general trend towards natural conditions up to three decades after rewetting

**Fig. 4:**
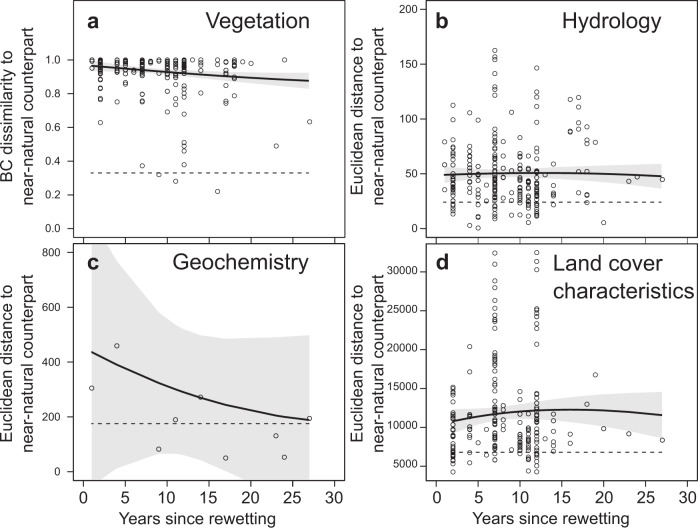
Strong differences and little to no temporal trend in the pairwise dissimilarity between rewetted sites and near-natural counterparts of similar origin. Dissimilarity in unitless Bray–Curtis (BC) dissimilarity for the vegetation (0 = identical species composition, 1 = no shared species) and in Euclidean distance (0 to infinite) for the other response clusters between each rewetted site with a near-natural counterpart (same peatland origination, comparable altitude, same biogeographic zone, minimum spatial distance) against the time since restoration. Black lines indicate a LOESS spline with span = 1.4 (lowest span not leading to uninformative multiple local minima or maxima) and its 95% confidence interval shaded in gray. Horizontal dashed lines indicate the mean dissimilarity between pairs of near-natural sites selected the same way as the near-natural counterparts of the rewetted sites. Linear models indicate highly significant intercepts (*p* < 0.001 for all four response clusters) and no significant slopes (*p* > 0.6) except for vegetation (*p* = 0.013).

A strong and, apparently, long-lasting difference in biodiversity, ecosystem functioning, and land cover characteristics between each rewetted site and its closest near-natural counterpart exists (Fig. 4). Even though 40% (vegetation) to 80% (hydrology) of the rewetted sites generally resembled composition or functioning of typical near-natural peatlands (falling into the 95% confidence ellipses in Fig. 2), the rewetted sites differed strongly from near-natural counterparts in biodiversity (vegetation), ecosystem functioning (hydrology, geochemistry) and land cover characteristics (linear models with *p*_*intercept*_ < 0.001 for all four response clusters despite low confidence due to small sample size for geochemistry). No detectable trend towards increasing similarity over time (linear models with *p*_*slope*_ > 0.05 for all four response clusters except vegetation with *p*_*slope*_ = 0.013 but still a predicted dissimilarity of 0.85 after 5 decades (95%CI: 0.64 to 1.07); Fig. 4) indicates that restoration success is either reached promptly after rewetting or not at all within the observed three decades covered by our data. Spatial variance exists also between near-natural counterparts of similar origin and distance, but at a much lower level than between rewetted and near-natural counterparts (horizontal dashed lines in Fig. 4 which is not included in the 95% confidence bands except for geochemistry with its low sample size). In short, time had little to no effect on dissimilarity between rewetted sites and their near-natural counterparts in our data, i.e., time seems to have little to no effect on restoration success for at least three decades.

It is commonly assumed that the stabilization of the water table close to the surface is a premise for successful restoration of peatlands^3^ because too low water tables lead to high carbon emissions and non-wetland plants remaining dominant while too high water tables create shallow lakes rather than peatlands. While closest similarity to near-natural counterparts with respect to biodiversity, ecosystem functioning and land cover characteristics indeed occurred for rewetted sites with annual median water tables close to the surface, we also observed sites with water tables close to the surface being strongly dissimilar to their near-natural counterparts. The general patterns indicate that restoration success depends on more than just a water table close to the surface (Supplementary Fig. 2).

### Locally novel ecosystems require functional understanding

With the existing dataset we cannot answer why some rewetted sites resemble near-natural counterparts right away, while others follow diverging trajectories for decades, indicating that the natural fens can become novel ecosystems *sensu* Hobbs et al.^41^, i.e., non-restorable at least for several decades, due to prolonged drainage (Fig. 4). Duration and intensity of drainage might likely matter here^3^ as these determine nutrient availability and irreversible changes in peat hydraulic variables (Fig. 4b). Existing knowledge from natural systems about ecological functions, external regulation, self-regulation, and ecosystem service provision appears hardly transferable for understanding and managing these locally novel ecosystems. Therefore, we call for a concerted action to assemble data about ecological functions and relevant meta information of rewetted peatlands as laid out by Bonnett et al.^42^. Understanding causes and consequences of restoration success will likely require the assessment of peat microbiomes and key microbial players in organic matter mineralization and GHG production and consumption and the GHG fluxes alongside vegetation, hydrology, pore water chemistry as well as chemical and physical peat properties.

Recently published studies showing that rewetted peatlands can act as carbon sinks even during extended dry and hot periods^43,44^ are encouraging, although the stop of continued carbon losses from peat degradation is the most pressing issue today^14^. However, historical drainage of peatlands followed by rewetting creates locally novel ecosystems in many cases. Understanding and management schemes cannot be transferred from natural systems. Instead, an interdisciplinary, process-based understanding of the rewetted systems is urgently needed to prioritize, plan and implement restoration measures and to design their sustainable management. The UN Decade of Ecosystem Restoration starting just now is the critical period for achieving our commitments of the Paris Agreement on peat soils^2^ and for peatland science.

## Methods

### Site selection

A total of 320 rewetted sites was compared to 243 undrained, near-natural sites (Supplementary Data 1), covering the major distribution range of temperate fens across Europe (Fig. 1). We included all sites where local experts confirmed a dateable rewetting action, usually the blocking of drainage systems, which led to a mean annual water table of 25 cm below surface or higher. Rewetting occurred on average 9 years (min. 1 and max. 54 years) before data sampling. Land use before rewetting was agriculture (80% of all rewetted sites), forestry (10%) or peat extraction (10%). We acknowledge that few, if any, untouched and completely natural temperate fens exist nowadays in Europe. For our comparison, we used sites without direct drainage history, again relying on local expert knowledge. We confirmed this by carefully checking field conditions and aerial/satellite imagery for structures which would imply substantial former drainage and disregarded those with such features. Our study contains some of the least disturbed fens in temperate Europe (Eastern Poland and Belarus).

### Response parameters

We included sites that provided data for at least two of the following four response clusters:

1. Vegetation: complete lists of vascular plants and bryophytes (539 species in total) based on 16 m^2^ (median, ranging between 12 and 25 m²) with estimates of individual plant species cover. All vegetation data collections included in this study aimed at full species lists and used comparable methodologies, i.e., estimating species-specific cover values. Studies focusing on specific taxa or just reporting the dominant species were excluded from the analyses. Mean species richness per plot was 15.1 (ranging from 2 to 63).
2. Hydrology: 269 piezometers with dataloggers, 91 piezometers related to a datalogger in a transect, 216 piezometers with manual measurements of at least one year and biweekly or monthly readings of the water table depth relative to the peat surface. The vast majority (>80%) of these datasets with only biweekly or monthly measurements were carried out for >2 years, so that we are confident in their power to estimate the temporal dynamics at the respective sites representatively. On average, 2.3 years were measured, and we included only data if available for at least one full year. All water table measurements have been made in direct vicinity to the vegetation relevée. We considered the annual median water table, annual minimum water table, annual maximum water table, and annual amplitude of water table (max minus min) as relevant response parameters. Data for water tables during the growing season were highly correlated to the annual water table data (annual median versus median throughout summer and autumn: *r*² = 0.89; annual median versus median throughout winter and spring: *r*² = 0.94; annual maximum versus maximum throughout summer and autumn: *r*² = 0.85; annual maximum versus maximum throughout winter and spring: *r*² = 0.86; annual minimum versus minimum throughout summer and autumn: *r*² = 0.91; annual minimum versus minimum throughout winter and spring: *r*² = 0.73).
3. Geochemistry: pH and electrical conductivity of the pore water (0–60 cm), bulk density and organic matter content of the top soil layer (0–30 cm) sampled in summer for all sites included here alongside the vegetation data sampling. The parameters considered for geochemistry and also for hydrology had to be chosen in order to maximize data availability in parallel with meaningfulness. Note that, still, geochemical data are only available for a subset of the full data set (57 sites; see Fig. 3e–h for sample size per group). Despite the low sample size, it is important to note that the remaining sites cover almost the whole geographical range of the other response parameters (Eastern Poland, Eastern and Northern Germany, the Netherlands, Belgium, Wales) and no geographical or ecological bias was found. In addition, the remaining sites contain well-comparable pairs of rewetted and near-natural sites throughout this geographical space. While the selected parameters allow for relevant insights, more specific response parameters are required for process-based understanding of the biogeochemistry of rewetted peatlands^42^.
4. Land cover characteristics: spectral-temporal metrics for a full annual time series of Copernicus Sentinel-2 A/B data for 2018. The Sentinel-2 A/B constellation provides optical imagery of the Earth’s surface between ~0.49–~2.2 µm in ten spectral bands and at 10–20 m ground sampling distance at a theoretical acquisition frequency of 2.5–5 days. We here acquired all available Sentinel-2 A/B imagery for 2018 with cloud cover <70% from the ESA API Hub. We used all valid observations to derive spectral-temporal metrics from the time series. Spectral temporal metrics are statistical measures (e.g., average, minimum, maximum, quartiles, …) per spectral band or index (e.g., *MNDWI* = (*green* *−* *short wave infrared*)/(*green* + *short wave infrared*)^45^) using all available cloud- and shadow-free observations over time. The median count of clear-sky-observations per pixel across the sites is 45, while 90% of all sites featured 27 clear-sky observations or more. Both data processing to Analysis Ready Data as well as calculating spectral-temporal metrics was performed through the Framework for Operational Radiometric Correction for Environmental monitoring^35^. Our analysis included data averaged over 3×3 pixels around the center plot location of the site. Different spatial aggregations (e.g., single pixels, 5×5 pixels around the center plot) led to highly similar results, implying that the intra-site variability was robust around locations of the vegetation survey. The inclusion of an annual series of Sentinel-1 synthetic aperture radar data (temporal metrics for VV and VH polarization, IW swath at 10 m resolution) for the same year did not affect the results.

The representativeness of our database is confirmed by the comparison to 10k vegetation plots from all over Europe. These have been randomly chosen from > 90k vegetation plots of the European Vegetation Archive^46^, classified as belonging to peatland types (Q2: “Poor fens”, Q4: “Calcareous rich fens”, Q5: “Tall helophyte beds”, Qa: “Mires”) of the EUNIS habitat types^18,28^. Plant taxa have been standardized to Euro + Med plantbase^47^ and the checklist of bryophytes of Europe^48^ and harmonized to exclude nested taxa (option maxtaxtable = ‘AG1’ and ag = ‘conflict’ in function taxval in R package vegdata^49^).

### Statistical analyses

An ordination (non-metrical multidimensional scaling, Bray–Curtis distance and step-across dissimilarities for those plot distances without common species with function ‘metaMDS’ from R-package ‘vegan’ 2.5-6) was applied in parallel to an analysis of similarity (function ‘anosim’ from package ‘vegan’ 2.5-6.) to test for significance of differences among rewetted and near-natural peatlands. An Indicator Species Analysis^50^ (function ‘indval’ from package ‘labdsv’ version 2.0-1) was applied to find plant species with significant affinity to rewetted or near-natural peatlands. NMDS ordinations and analyses of similarity based on Euclidean distance were calculated for the other three response clusters, i.e., hydrology, geochemistry, and land cover characteristics. Variance, quantified as pairwise distance in the response clusters, was compared between rewetted and near-natural sites by testing for significance of differences in mean values per group. As data points in pairwise dissimilarity matrices are not strictly independent^51^ and homoscedasticity and normal distribution of residuals were questionable, we relied on permutation based procedures for estimating the significance of differences in mean values of pairwise dissimilarities between groups^51^ (function ‘diffmean’ from package ‘simba’ version 0.3-5 with 1000 permutations per test).

In order to account for large variance in the predictors due to the broad spatial coverage of our database, additional analyses relied on pairwise comparisons between rewetted sites and near-natural counterparts. All sites were classified according to their biogeographic peatland region and hydrogenetic and ecological fen type according to Joosten et al.^10^. Then, the spatially closest near-natural counterpart was assigned to each rewetted site (Supplementary Data 1). If no counterpart within the class was available, the site was disregarded for the pairwise analysis. For sites from mountainous regions, mainly southern Germany, counterparts also had to stem from similar altitudes and sites with no near-natural counterpart within the same class and at similar altitude were disregarded. For comparison, near-natural-to-near-natural pairs were built using the same methodology and mean near-natural-to-near-natural dissimilarity is displayed in Fig. 4 as horizontal dashed line. Mean distance between rewetted-to-natural counterparts were 9, 40, 11, and 21 km for vegetation, hydrology, geochemistry and land cover characteristics, respectively, whereas near-natural-to-near-natural pairs were 8, 20, 21, and 11 km apart from each other on average.

### Reporting summary

Further information on research design is available in the Nature Research Reporting Summary linked to this article.

## Supplementary information

Supplementary Information File

Description of Additional Supplementary Files

Dataset 1

Dataset 2

Reporting Summary

## Data Availability

The data generated and analyzed in this study have been deposited in the Dryad database under 10.5061/dryad.08kprr532. The plot-based vegetation data used for comparison to this dataset is available at the European Vegetation Archive^46^.

## Code availability

All analyses were carried out with the functions and additional packages specified in the methods section in the free and open-source environment R^52^.
