# Supplementary material for: Rewetting does not return drained fen peatlands to their old selves
Source: Nat Commun. 2021 Oct 5;12:5693. doi: 10.1038/s41467-021-25619-y (PMC8492760; doi:10.1038/s41467-021-25619-y)
Supplement: Supplementary file 1 — Supplementary Information File [file 41467_2021_25619_MOESM1_ESM.pdf]

Supplementary Information file to

Rewetting does not return drained fen peatlands to their old selves

Kreyling J*, Tanneberger F, Jansen F, van der Linden S, Aggenbach C, Blüml V, Couwenberg J, Emsens W-J, Joosten H, Klimkowska A, Kotowski W, Kozub L, Lennartz B, Liczner Y, Liu H, Michaelis D, Oehmke C, Parakenings K, Pleyl E, Poyda A, Raabe S, Röhl M, Rücker K, Schneider A, Schrautzer J, Schröder C, Schug F, Seeber E, Thiel F, Thiele S, Tiemeyer B, Timmermann T, Urich T, van Diggelen R, Vegelin K, Verbruggen E, Wilmking M, Wrage-Mönnig N, Wołejko L, Zak D, Jurasinski G

Corresponding author email: Juergen.kreyling@uni-greifswald.de

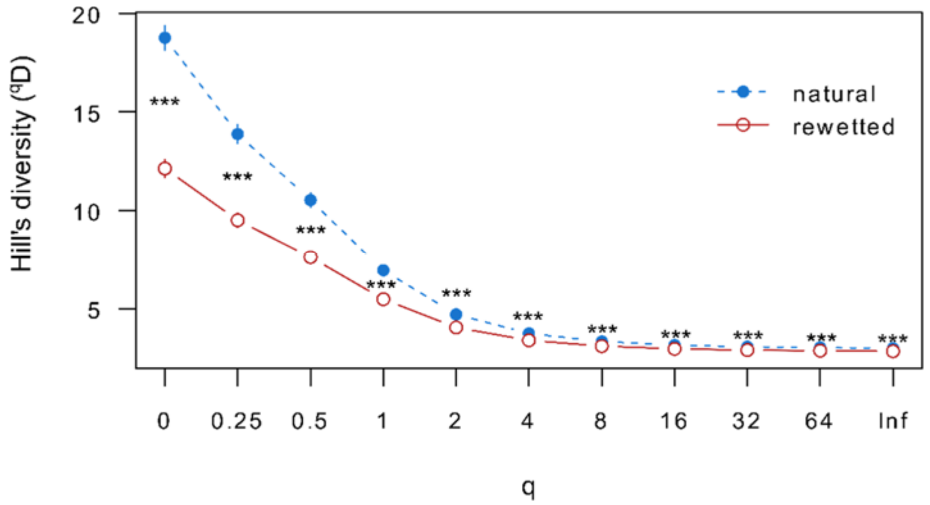

Supplementary Figure 1 | Hill's diversity numbers[1] compared between 320 rewetted and 243 natural sites. At q=1 the species contribute with their true abundances to the mathematical result, below 1 rare species increase their influence on the result, above 1 the importance of common species increases. All pairwise comparisons between rewetted and near-natural were highly significant (***: $p < 0.001$) according to post-hoc tests (function 'emmeans' of the package 'emmeans' 1.5.1) of a two-sided mixed model ANOVA with ${}^{q}D$ as response parameter, q and the drainage status (rewetted vs. near-natural) as fixed effects and siteID as random effect to account for dependencies in the data. According to the Analysis of Variance of the mixed model, the two fixed effects and their interaction yielded significance ($p < 0.001$). Hill's diversity was calculated with the function 'renyi' of the package 'vegan' 2.5-6 in R[2]

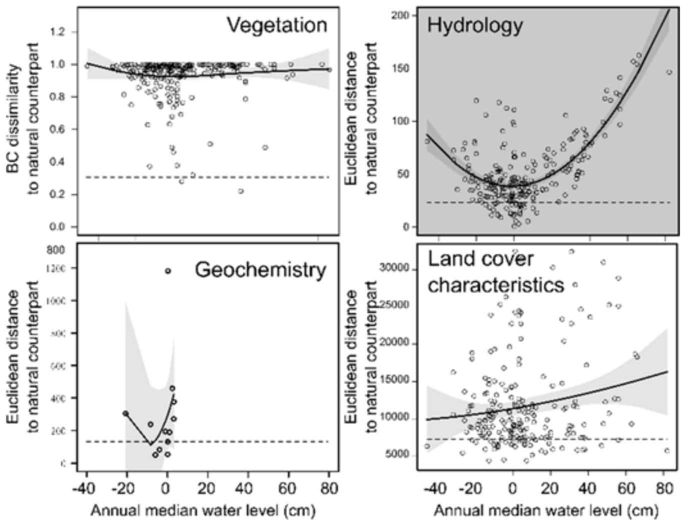

Supplementary Figure 2 | Water table close to surface has little effect on the pairwise dissimilarity between rewetted sites and natural counterparts of similar origin. Dissimilarity in unitless Bray Curtis dissimilarity for the vegetation (0 = identical species composition, 1 = no shared species) and in Euclidean distance (0 to infinite) for the other response clusters between each rewetted site with a natural counterpart (same peatland origination, comparable altitude, same biogeographic zone, minimum spatial distance) against median annual water table of the rewetted sites. Black lines indicate a LOESS spline with span =1.4 (lowest span not leading to uninformative multiple local minima or maxima) and its 95% confidence interval shaded in grey. Horizontal dashed lines indicate the mean pairwise dissimilarity of semi-natural to semi-natural counterparts selected the same way as the semi-natural counterparts of the rewetted sites. Linear models indicate highly significant intercepts ($p < 0.001$ for all four response clusters) and no significant slopes ($p = 0.158$ and $p = 0.159$) for vegetation and geochemistry. The results for hydrology are inconclusive and therefore shaded in grey as the mean annual water table (x-axis) is also part of the input variables used to derive the Euclidean distance (y-axis). A significant positive relationship for land cover characteristics ($p = 0.004$) was detected by the linear model.

Supplementary Table 1 | Relative frequency of 320 rewetted and 243 semi-natural fen peatlands attributable to EUNIS 2020 habitats[3,4]. Sorted by decreasing absolute difference in the relative frequency between natural and rewetted peatlands. The higher relative frequency is set off in bold for all absolute differences larger than 5%. Chi² test: $p < 0.0001$. Note that some natural sites were classified into EUNIS habitats commonly not considered fens. We conservatively kept those in the analyses in order to avoid an artificial bias that would reduce variation among the natural sites.

EUNIS 2020	natural	rewetted	EUNIS 2020 habitat name
Q51	6.2	**30.5**	Tall-helophyte bed
Qb	**25.5**	5.6	Wetlands
Qa	**7.5**	0.3	Mires
R	7.5	**14.1**	Grasslands and lands dominated by forbs, mosses or lichens
R36	0.0	4.5	Moist or wet mesotrophic to eutrophic pasture
R37	4.2	0.0	Temperate and boreal moist or wet oligotrophic grassland
C12b	0.0	4.0	Rooted submerged vegetation of oligotrophic waterbodies
Q53	2.9	6.1	Tall-sedge bed
Q24	3.3	0.3	Intermediate fen and soft-water spring mire
Q42	2.6	0.0	Extremely rich moss–sedge fen
C	0.0	2.4	Inland surface waters
Q22	2.3	0.0	Poor fen
S92	3.3	1.1	Salix fen scrub
C35a	0.0	1.9	Plankton communities of eutrophic standing waters
Q52	1.6	3.2	Small-helophyte bed
R35	4.9	6.4	Moist or wet mesotrophic to eutrophic hay meadow
Q41	1.0	0.0	Alkaline, calcareous, carbonate-rich small-sedge spring fen
R21	0.0	0.8	Mesic permanent pasture of lowlands and mountains
T	0.0	0.8	Forests and other wooded land
Q43	0.7	0.0	Tall-sedge base-rich fen
T15	0.7	0.0	Broadleaved swamp forest on non-acid peat
R55	0.3	0.8	Lowland moist or wet tall-herb and fern fringe
T12	0.3	0.0	Alnus glutinosa–Alnus incana forest on riparian and mineral soils
T16	0.3	0.0	Broadleaved mire forest on acid peat
H25	0.0	0.3	Acid siliceous screes of warm exposures
R22	0.7	0.5	Low and medium altitude hay meadow

Supplementary Table 2 | r^2 for all significant ($p < 0.05$) spectral-temporal metrics of the ordination in Figure 1.

Metric	r^2	Metric	r^2	Metric	r^2	Metric	r^2	Metric	r^2
MNW_AVG	0.89	TCW_AVG	0.72	MNW_STD	0.58	BLU_MAX	0.30	RED_SKW	0.11
MNW_Q75	0.88	NDV_Q75	0.72	TCW_MAX	0.57	NDV_IQR	0.30	RE3_SKW	0.11
MNW_Q50	0.87	NDV_Q25	0.72	NDV_RNG	0.55	RED_Q90	0.30	BNR_SKW	0.11
NIR_Q75	0.86	NDV_MAX	0.71	GRN_Q50	0.55	BLU_MIN	0.29	RE2_SKW	0.10
NIR_AVG	0.86	MNW_Q10	0.71	TCG_RNG	0.54	GRN_Q90	0.29	GRN_IQR	0.10
TCB_Q50	0.83	TCW_Q75	0.71	SW2_Q90	0.54	NIR_STD	0.29	BLU_IQR	0.10
MNW_MAX	0.83	TCB_Q10	0.70	NDV_Q50	0.53	SW1_RNG	0.28	NDB_IQR	0.09
BNR_AVG	0.83	TCB_MAX	0.70	SW2_MAX	0.51	TCB_IQR	0.27	NDB_SKW	0.09
BNR_Q75	0.83	NDV_Q90	0.70	GRN_AVG	0.51	BLU_RNG	0.27	RE1_IQR	0.08
NIR_Q90	0.83	TCW_Q50	0.70	GRN_MIN	0.50	SW1_STD	0.27	SW1_KRT	0.08
MNW_Q90	0.83	BNR_Q25	0.69	MNW_IQR	0.49	TCG_IQR	0.27	RED_KRT	0.07
TCG_AVG	0.82	NIR_Q10	0.69	NDV_STD	0.46	BNR_STD	0.27	RE1_SKW	0.05
TCB_Q75	0.82	SW2_Q10	0.69	RE1_Q75	0.43	SW1_IQR	0.26	NDB_Q90	0.05
NDV_MIN	0.82	SW1_Q10	0.68	TCG_STD	0.42	RED_STD	0.25	NDB_MAX	0.04
RE3_AVG	0.82	RE3_Q25	0.68	NDB_MIN	0.41	RE3_STD	0.25	RE3_KRT	0.04
MNW_Q25	0.82	SW1_Q90	0.68	TCW_RNG	0.41	NDB_AVG	0.24	TCB_KRT	0.04
RE3_Q75	0.82	RE2_Q25	0.68	TCB_RNG	0.40	GRN_STD	0.23	TCG_KRT	0.04
TCG_Q75	0.81	RE1_Q10	0.67	RED_AVG	0.39	BLU_Q75	0.23	RE2_KRT	0.03
TCB_AVG	0.81	TCW_Q25	0.67	RE1_MAX	0.39	RE2_STD	0.23	NIR_KRT	0.03
NIR_MAX	0.81	MNW_MIN	0.67	BLU_Q25	0.39	NDB_Q50	0.22	BNR_KRT	0.03
NDV_Q10	0.80	BNR_Q10	0.67	BLU_Q10	0.38	BLU_STD	0.21	NDV_KRT	0.03
SW1_Q50	0.80	RE1_Q25	0.66	TCW_STD	0.38	SW2_RNG	0.21	TCG_SKW	0.02
NIR_Q50	0.80	TCW_Q90	0.66	RED_Q25	0.38	BLU_Q90	0.20	NDV_SKW	0.02
RE2_Q75	0.80	TCG_Q50	0.66	GRN_Q75	0.37	RE1_STD	0.20	NDB_KRT	0.00
RE2_AVG	0.80	RE3_Q10	0.65	TCB_STD	0.37	NDB_STD	0.20		
NDV_AVG	0.80	SW2_Q75	0.65	NIR_RNG	0.37	RED_IQR	0.19		
SW1_AVG	0.79	SW1_MAX	0.65	RED_Q50	0.37	SW2_STD	0.18		
BNR_Q90	0.79	NIR_MIN	0.64	GRN_MAX	0.37	SW2_IQR	0.18		
TCG_MAX	0.79	RE2_Q10	0.64	RED_MAX	0.36	SW1_SKW	0.18		
TCG_Q90	0.79	TCB_MIN	0.64	BNR_RNG	0.35	SW2_SKW	0.18		
BNR_MAX	0.77	BNR_MIN	0.62	RED_MIN	0.35	NIR_IQR	0.17		
RE3_Q90	0.77	MNW_RNG	0.62	RE1_Q90	0.35	GRN_KRT	0.16		
BNR_Q50	0.76	RE1_Q50	0.61	BLU_Q50	0.35	BNR_IQR	0.15		
SW1_Q75	0.76	SW2_MIN	0.61	NDB_Q25	0.35	NIR_SKW	0.14		
RE3_MAX	0.75	TCW_Q10	0.61	BLU_AVG	0.35	SW2_KRT	0.14		
RE2_Q90	0.75	RE3_MIN	0.61	RE3_RNG	0.34	RE3_IQR	0.14		
TCB_Q90	0.75	SW1_MIN	0.60	TCW_IQR	0.33	RE1_KRT	0.14		
TCB_Q25	0.74	TCG_Q10	0.60	NDB_Q10	0.33	TCB_SKW	0.13		
SW1_Q25	0.74	RE2_MIN	0.60	RED_RNG	0.33	BLU_KRT	0.13		
RE3_Q50	0.74	GRN_Q10	0.59	RED_Q75	0.32	TCW_SKW	0.12		
RE2_MAX	0.73	TCG_Q25	0.59	NDB_RNG	0.32	GRN_SKW	0.12		
SW2_Q25	0.73	RE1_MIN	0.59	GRN_RNG	0.32	MNW_KRT	0.12		
RE2_Q50	0.73	TCG_MIN	0.58	RE2_RNG	0.31	BLU_SKW	0.12		
SW2_AVG	0.73	TCW_MIN	0.58	RE1_RNG	0.30	NDB_Q75	0.12		
SW2_Q50	0.73	RE1_AVG	0.58	RED_Q10	0.30	RE2_IQR	0.11		
NIR_Q25	0.72	GRN_Q25	0.58	MNW_SKW	0.30	TCW_KRT	0.11		

Sentinel-2 reflectance bands: BLU – blue (0.492 µm), GRN – green (0.559 µm), RED – red (0.665 µm), RE1 – red edge 1 (0.704 µm), RE2 – red edge 2 (0.740 µm), RE3 – red edge 3 (0.783 µm), NIR - near infrared (0.833 µm), BNR – broad near infrared (0.864 µm), SW1 – short wave infrared 1 (1.61 µm), SW2 – short wave infrared 2 (2.19 µm); spectral indices: MNW – modified normalized difference water index[5], NDV – Normalized difference vegetation index[6], NBR – normalized burn ratio[7], TCB/TCG/TCW – tasseled cap brightness/greenness/wetness[8]; metrics: AVG – average, STD – standard deviation, SKW – skewness, KRT – kurtosis, MIN – minimum, MAX – maximum, RNG – range, Q10/25/50/75/90– percentiles (10, 25, 50, 75, 90), IQR – inter-quartile range.

Supplementary References

1. Hill, M. O. Diversity and evenness: a unifying notation and its consequences. *Ecology* **54,** 427–432; 10.2307/1934352 (1973).
2. R Core Team. *R: A Language and Environment for Statistical Computing. R version 4.0.2* (R Foundation for Statistical Computing. URL http://www.R-project.org, Vienna, Austria, 2020).
3. Bruelheide, H., Tichý, L., Chytrý, M. & Jansen, F. Implementing the formal language of the vegetation classification expert systems (ESy) in the statistical computing environment R. *Appl Veg Sci* **24**; 10.1111/avsc.12562 (2021).
4. Chytrý, M. *et al.* EUNIS Habitat Classification: Expert system, characteristic species combinations and distribution maps of European habitats. *Appl Veg Sci* **23,** 648–675; 10.1111/avsc.12519 (2020).
5. Xu, H. Modification of normalised difference water index (NDWI) to enhance open water features in remotely sensed imagery. *Int J Remote Sens* **27,** 3025–3033; 10.1080/01431160600589179 (2006).
6. Tucker, C. J. Red and photographic infrared linear combinations for monitoring vegetation. *Remote Sens Environ* **8,** 127–150; 10.1016/0034-4257(79)90013-0 (1979).
7. Roy, D. P., Boschetti, L. & Trigg, S. N. Remote sensing of fire severity: assessing the performance of the Normalized Burn Ratio. *IEEE Geosci. Remote Sensing Lett.* **3,** 112–116; 10.1109/LGRS.2005.858485 (2006).
8. Shi, T. & Xu, H. Derivation of tasseled cap transformation coefficients for Sentinel-2 MSI At-sensor reflectance data. *IEEE J. Sel. Top. Appl. Earth Observations Remote Sensing* **12,** 4038–4048; 10.1109/JSTARS.2019.2938388 (2019).
