# Supplementary material for: Rewetting does not return drained fen peatlands to their old selves
Source: Nat Commun. 2021 Oct 5;12:5693. doi: 10.1038/s41467-021-25619-y (PMC8492760; doi:10.1038/s41467-021-25619-y)
Supplement: Supplementary file 2 — Description of Additional Supplementary Files [file 41467_2021_25619_MOESM2_ESM.pdf]

Description of Additional Supplementary Information

Title: Supplementary Data 1

Description: All study sites with their coordinates (decimal degrees with ',' as decimal), ID of the near-natural counterpart for the rewetted sites or the near-natural pair for the near-natural sites and indication of data availability in the four response clusters, sorted by data availability and increasing longitude.

Title: Supplementary Data 2

Description: Plant species with significant preference for rewetted or near-natural fen peatlands according to an Indicator Species Analysis. Plants are ordered by decreasing indicator value with indicator values for natural and rewetted peatlands provided as numbers together with their significance.
